# An investigation of the potential of rapid prototyping technology for image‐guided surgery

**DOI:** 10.1120/jacmp.v7i4.2302

**Published:** 2006-11-28

**Authors:** Didier A. Rajon, Frank J. Bova, R. Rick Bhasin, William A. Friedman

**Affiliations:** ^1^ Neurosurgery Department University of Florida Gainesville Florida 32610 U.S.A

**Keywords:** rapid prototyping, image‐guided surgery, stereotactic frame

## Abstract

Image‐guided surgery can be broken down into two broad categories: frame‐based guidance and frameless guidance. In order to reduce both the invasive nature of stereotactic guidance and the cost in equipment and time, we have developed a new guidance technique based on rapid prototyping (RP) technology. This new system first builds a computer model of the patient anatomy and then fabricates a physical reference frame that provides a precise and unique fit to the patient anatomy. This frame incorporates a means of guiding the surgeon along a preplanned surgical trajectory. This process involves (1) obtaining a high‐resolution CT or MR scan, (2) building a computer model of the region of interest, (3) developing a surgical plan and physical guide, (4) designing a frame with a unique fit to the patient's anatomy with a physical linkage to the surgical guide, and (5) fabricating the frame using an RP unit. Software was developed to support these processes. To test the accuracy of this process, we first scanned and reproduced a plastic phantom fabricated to validate the system's ability to build an accurate virtual model. A target on the phantom was then identified, a surgical approach planned, a surgical guide designed, and the accuracy and precision of guiding a probe to that target were determined. Steps 1 through 5 were also evaluated using a head phantom. The results show that the RP technology can replicate an object from CT scans with submillimeter resolution. The fabricated reference frames, when positioned on the surface of the phantom and used to guide a surgical probe, can position the probe tip with an accuracy of 1.7 mm at the probe tip. These results demonstrate that the RP technology can be used for the fabrication of customized positioning frames for use in image‐guided surgery.

PACS number: 87.57.Gg

## I. INTRODUCTION

The ability to map the brain into a set of 3D coordinates is the fundamental basis of stereotactic neurosurgery. Translating this map into a fixed frame system or a frameless optical system facilitates minimally invasive, accurate, and safe surgery. This technique is used on a daily basis by neurosurgeons to perform brain biopsies, tumor resections, ventricular shunt placements, deep brain stimulator placements, ablative neurosurgery, and spinal instrumentation.

The development of X‐ray CT and MRI has revolutionized the neurosurgeon's ability to plan and execute surgery. To aid the surgeon in appreciating the location of target tissues, computer workstations are used to create 3D models and three‐plane views of intracranial anatomy. This allows the surgeon to test and manipulate alternative paths of approach to the target. This type of image‐guided surgery falls into two broad categories: frame‐based guidance and frameless optical guidance.

There are several limitations of both types of guidance. Frame‐based systems involve a head ring being attached to the patient. This can be uncomfortable and requires technical expertise. The patient must have an imaging study with the ring in place to provide the localization. Thus this must occur the morning of surgery. Once in the operating room, the scan must be reviewed. Planning of entry, trajectory, and target must occur while the patient is waiting. All these steps add a significant amount of both anesthesia and operating room time to the procedure. The same‐day scan inherently limits the ability to perform any type of fixed preplanning.

Frameless image guidance systems have their limitations as well. The patient may need fiducial markers placed on the head, and a scan, either CT or MRI, needs to be performed prior to surgery. This can be performed the day prior to surgery; however, it is typically performed the morning of surgery. Once in the operating room, the head needs to be fixated to the operating table with a clamp, and the frameless navigation system needs to be calibrated, tested, and planned. This takes a significant amount of operating room and anesthesia time, during which no actual incision is made. Another major limitation of frameless navigation systems is cost. Modern units cost close to a half a million dollars, and many hospitals are unable to justify the purchase of such systems. In addition, personnel must be hired who can operate, maintain, and troubleshoot these complex systems.

To address these issues, alternatives to the frame‐based and frameless stereotactic systems are being pursued. An ideal system would allow the surgeon to plan the surgery the day the films are first reviewed, involve a system with minimal operating room and anesthesia time, provide minimal discomfort to the patient, and provide at least the same accuracy as current systems. All this must be done at a minimal cost with a minimal number of technical personnel.

Any new system must provide an acceptable accuracy and precision equivalent or superior to currently available technologies. The gold standard for stereotactic accuracy is the frame‐based stereotactic system. The overall accuracy of such a system is determined by a convolution of imaging accuracy and the system's mechanical accuracy. For commercially available systems this accuracy has been determined to be 3.68 mm when 0.67 mm×0.67 mm in‐plane pixels were obtained and 1.0‐mm slice thickness were used. The accuracy extrapolated for zero slice thickness was determined to be 2.28 mm.^(^ ^1^ ^,^ ^2^ ^)^

Over the past five years, advances in the field of rapid prototyping (RP) equipment have provided an opportunity to design and develop guidance systems that can provide the benefits of stereotactic guidance with minimal limitations. Rapid prototyping, also known as solid free‐form fabrication, is the automatic construction of physical objects using 3D printers, stereolithography machines, or selective laser sintering systems. RP is a type of computer‐aided manufacturing and is one of the components of the rapid manufacturing process. This technology has existed in the industrial world since the 1980s, with machines used to design consumer products, buildings, and automobiles. Traditionally RP technology has been used to produce physical prototypes; however, recent advances have made the technology cheaper and more accessible, allowing the actual production of tools and parts.

Applying RP technology to image‐guided surgery is a hybrid of frame and frameless guidance. The frameless portion involves registering the 3D intracranial volume using fixed external landmarks: anatomical landmarks or fiducial markers placed on the patient's skin. Each landmark is first localized on the computer‐generated virtual model of the patient's head. It is then localized on the patient using the navigation system. The two sets of landmarks are then merged by the computer to correlate the patient with the virtual model. This step involves a minimum number of landmarks, usually 8 or 10. In essence, the computer workstation is able to create a rough facial mask from the landmark points and merge the 3D volume into that mask using best‐fit calculations. A rapid prototyping solution to this problem is to create an actual mask based on the 3D volume that can be placed on the patient at the time of surgery. This is the frame portion of this technology, since the mask is a physical object and provides the surgeon with a clear understanding of how well the computer‐generated volume fits the actual patient.

The goal of this project was to first develop software that provided the surgeon with the ability to build a virtual 3D model from a diagnostic image dataset and to then plan an intracranial surgical approach. The software allows the surgeon to fabricate a patient‐specific reference frame with RP technology. This reference frame could incorporate all necessary trajectories for guidance, including mechanical referencing to the patient at the time of surgery, guidance for the initial skin incision, position and design of the required craniotomy or craniectomy, and trajectory alignment to the target tissues. The specific aim of the work is to evaluate the user interface used to build and design the guide mold and the effect of user‐defined parameters, such as segmentation levels, on overall system accuracy.

## II. METHODS

RP is a technology that takes a 3D computer model and fabricates a 3D object by building layers upon layers of material.^(^ ^3^ ^–^ ^6^ ^)^ As a standard, the computer model is written in stereolithography (STL) format. The STL format represents a 3D surface as an assembly of planar triangles. A preprocessing software program slices the STL model into a number of layers from 0.01 mm to 0.7 mm thick, depending on the RP technique. Then the object is built one layer at a time, from bottom to top, each layer becoming the support surface of the following one. Depending on the technique, overhangs or undercuts may not be supported by the previous layer alone and may require a support structure to be deposited around the object. The most common techniques of fabrication are STL, laminated object manufacturing, selective laser sintering, fused deposition modeling, solid ground curing, and ink‐jet printing.^(^ ^7^ ^–^ ^12^ ^)^

**Figure 1:**
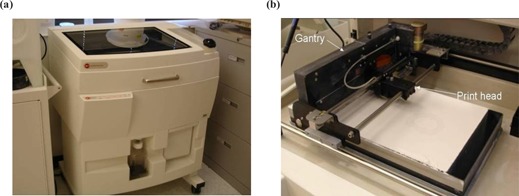
3D printer (ZCorp, model 310) used to fabricate the reference frames. (a) General view of the printer. (b) The inside of the printer showing the print head and the gantry

**Figure 2:**
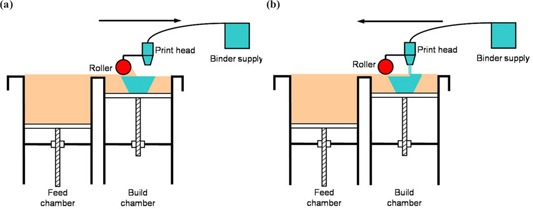
Scheme showing the principle of 3D printing. (a) A layer of powder is spread over the top of the build chamber. (b) On its way back to the feed chamber, the print head deposits the liquid binder to form a cross section of the object. Note that the lateral translation of the print head is not shown.

We chose to use a 3D printer for this project. The rapid fabrication time, the low price of supplies, and the ability to subject the final product to gas sterilization were all factors in our decision. Three‐dimensional printers (3DP) are part of a larger class of machines that use ink‐jet technology.^(^ ^7^ ^,^ ^12^ ^)^ In this technology, a layer of powdered material is selectively fused by a binder fluid deposited, or “printed,” by an ink‐jet printing head (Figs. 1(a) and 1(b)). Unbound powder remains to support the next layer. The unbound powder is blown off at end of built. The current price for most 3DPs is about $20 000. We chose the system manufactured by ZCorp, model 310 (Burlington, MA). This system provided a build specification of 0.3 mm and a build bin that can accommodate a part that is 25 cm×25 cm×20 cm. The system is composed of two chambers: the feed chamber, which is filled with powder at the start, and the build chamber, which is empty (Fig. 2). A gantry is allowed to move horizontally over both chambers. It carries a print head and a roller. The print head is fed by gravity from the binder supply. The roller is used to transfer the powder from the feed chamber to the build chamber. Before each layer, the feed chamber is raised by 0.3 mm, whereas the build chamber is lowered by 0.3 mm. In Fig. 2(a) the gantry is translated toward the right, and the roller pushes and compresses the powder on top of the build chamber. The print head is inactive during this phase. In Fig. 2(b), the gantry moves toward the left. The roller has no effect, and the print head is active. As the gantry moves, the print head sweeps the surface perpendicular to the translation axis and fuses the powder by depositing the fluid binder. Then the chambers are moved vertically again, and a new cycle can start. At the end of fabrication the object is removed from the build chamber, the excess powder is blown off the object, and the surface is infiltrated with wax, cyanoacrylate glue, or other sealants to improve durability and surface finish. When the infiltrant is dry, the object is ready for use. This can be a nonsterile fixture or subjected to gas sterilization for use within a sterile operative field.

**Figure 3:**
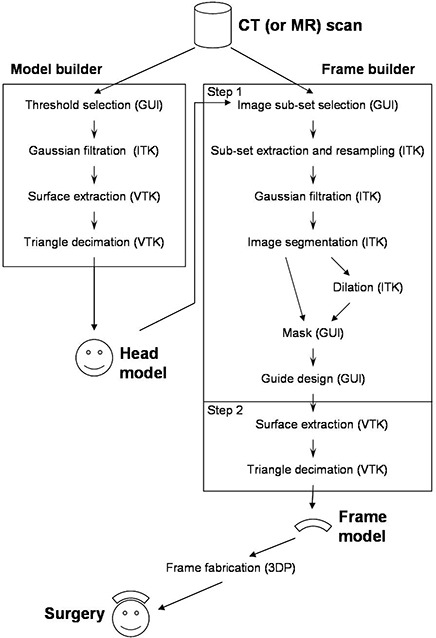
Flowchart of the different components of the program developed for designing the custom positioning frame. For each step, the acronym in parentheses represents the tool used to perform the step: ITK, VTK, 3DP (physical fabrication by the RP machine), and GUI (our own development or user intervention).

To investigate the capabilities of RP technology for image‐guided surgery, it was decided to first evaluate the possibility of reproducing a feature of the patient anatomy from the image dataset. A graphic user interface (GUI) was developed that provided image‐processing capabilities to support the construction of virtual 3D models, reference frame, and guide. The program was based on existing toolkits. The Insight Toolkit (ITK 1.8, Kitware Inc., Clifton Park, NY) is an image‐processing software program designed mainly for image segmentation and image registration. The Visualization Toolkit (VTK 4.2, Kitware Inc., Clifton Park, NY) is dedicated to 3D rendering and can work directly on image datasets or on 3D models such as polygonal surfaces. Although VTK includes some image‐processing features, it is recommended to use ITK for image processing and to interface the processed image with VTK for rendering. Both ITK and VTK are open source software programs distributed by Kitware, Inc. The user interface was developed using Qt (Qt 3.3.3, Trolltech Inc., Oslo, Norway), a software program distributed by Trolltech. All three software programs are written in C++ and are object‐oriented, which facilitates their cohabitation within a common application. All other necessary code was also developed using C++. A flowchart of the different components of our program is presented in Fig. 3. The reader can refer to this figure while reading the different parts of our study.

### A. Fabrication of a replica of a tissue‐like phantom

**Figure 4:**
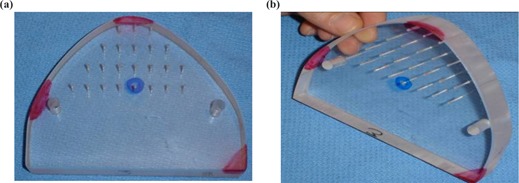
Two different views of the lexan phantom used for our study. (a) The front side is shown with the target indicated by a blue circle. (b) The phantom is seen from a different angle to show its thickness. The red areas indicate the three regions that were chosen to position the three different frames.

The phantom used for initial testing is shown in Fig. 4. It is made of lexan, which produces an image with tissue‐like properties when imaged using a diagnostic CT scanner. The density of lexan is $1.19\;g/{\text{cm}}^{3}$. CT scans of the phantom were obtained using a Siemens Sensation 16 scanner. A head reconstruction protocol, an in‐plane resolution of 0.35 mm, and a slice thickness of 0.75 mm were used. For ease of measurements, we chose a phantom that had two flat surfaces. For CT scan acquisition we tilted the object at approximately 45± from the image planes to avoid any alignment of the images with the flat surfaces, thereby avoiding a false creation of ideal planar surfaces during 3D reconstruction. A series of holes was drilled through the phantom. The intersection of the center hole and the phantom's surface, surrounded by a blue circle in Fig. 4, was used as our target feature.

**Figure 5:**
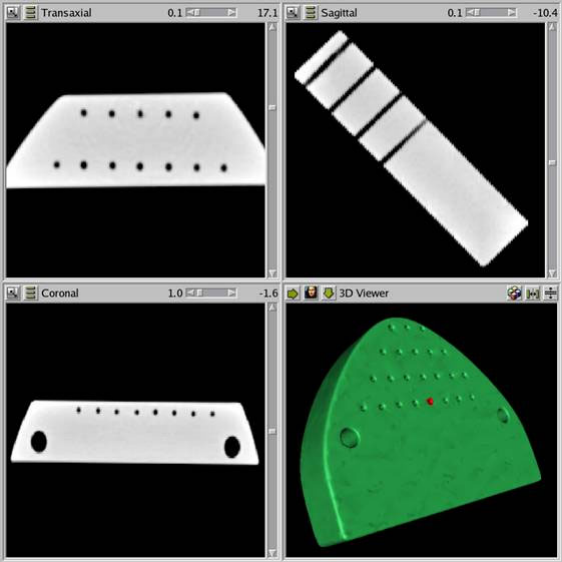
A snapshot of the graphic user interface. It shows the three orthogonal views of the image dataset of the lexan phantom after 3D reconstruction. It also shows the 3D rendering of the model built from the image dataset. The red spot indicates the surgical target selected for the current study.

The DICOM images were later transferred to a local server and processed into a format compatible with volumetric data for use in ITK and VTK. The data are initially displayed in standard three orthogonal view format, as shown in Fig. 5.

The next step was to build a 3D model. This was done by a series of image‐processing steps. These steps are summarized in the block “Model builder” of the flowchart in Fig. 3. The image was segmented using a threshold (discussed later) that splits the image voxels into two groups: the voxels inside the phantom and the voxels outside the phantom. This simple segmentation technique has proven to be effective for CT‐based datasets and many MR datasets. The image data were passed through a Gaussian filter with a standard deviation of 0.4 mm to reduce noise. The virtual boundary of the phantom was extracted from this processed image using the Marching Cube algorithm.^(13)^ The output is a set of triangles that are connected together to form a closed surface that conforms to the object boundary. The set of triangles was then decimated to reduce the number of triangles. The decimation is performed by merging all adjacent triangles which alignment is within a certain angle. The value of 4± was retained as the maximum angle and can routinely provide a reduction factor of 0.1 (tenfold reduction) without affecting the rendering. Note that since the decimation process is based on the angle between triangles, the small details of the surface are preserved, because the triangles of such small details retain their original size (i.e., the initial CT scan resolution). For larger details, hundreds of triangles belonging to the same flat surface may be merged into just a few ones. This technique thus preserves the topology of the extracted surface. Both the value of the standard deviation of the Gaussian filter and the maximum angle for merging the triangles are dependent on the scanning device. A series of trials with visual inspection of the resulting 3D rendering allowed determination of the two values mentioned above for our specific CT scanner. When performing the trials, our goal was to smooth the image in order to reproduce the aspect of the physical object while preventing the surface from being shifted from its position before smoothing. The shift can easily be assessed on a 2D cross section of the image with the curve (cross section of the rendered surface) placed on top. When most CT scanners require a standard deviation about the size of the in‐plane pixel resolution (0.4 vs 0.35 in our case), MR images necessitate a larger Gaussian kernel, up to 2.5 times the pixel size. Besides the choice of the threshold (discussed next), the image‐processing steps, if performed carefully, should introduce little error compared to the image resolution. The virtual model with identified target is shown in Fig. 5.

In order to properly build a model from the medical scan data, the system must have the ability to threshold the data with sufficient spatial resolution to allow an unambiguous positive of the surface to be fabricated. While the ultimate test of such a system is the ability to accurately threshold the patient's skin, the nonrigid nature of skin proves to be a limiting factor. It was decided that a more appropriate set of tests could be carried out using a phantom of near tissue equivalence that presented a rigid surface against which accurate dimensions could be obtained. To test the segmentation technique, we built several models with different threshold values. Because of the density of lexan $\left(1.19\;g/{\text{cm}}^{3}\right)$ the CT numbers inside the phantom are around 1200. As a consequence, the selected thresholds were 200, 400, 600, 800, and 1000, which cover the full range from air to lexan. The five models were converted into an STL format and sent to our RP machine for fabrication.

**Figure 6:**
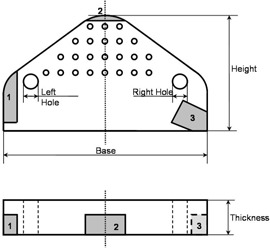
Scheme of the phantom showing the features chosen for the geometric measurements of the replica. The shaded regions represent the surfaces of the phantom that were chosen for positioning the frames. They are labeled “1,” “2,” and “3.” “Base” is the length of the phantom along its longest dimension. “Height” is the maximum height between the base and the top of the phantom. “Thickness” is measured from the front side to the back side. “Left hole” and “Right hole” are the diameters of the two large holes measured along a direction parallel to the base.

Five features of the phantom were measured for assessment of the replication accuracy. The five features are shown in Fig. 6.

### B. Defining surgical paths

In order to build a surgical guide, a reference surface must be identified. For this surface to provide an unambiguous reference, or fit, the surface must have unique geometric features. Figure 6 shows three surfaces that were designed as potential surfaces for reference:

- Surface 1 is on a section of the phantom that has a gentle slope. This reference does not provide a particularly unique fit, with the surface appearing to align in several different positions along the object's surface. Each position will provide an equal feeling of stability for an experimenter trying to position the frame on the surface.
- Surface 2 is at the apex of the phantom. While this provides a more unique fit, it still has some ambiguity when aligned by the user.
- Surface 3 includes the corner of the phantom and provides a very unique fit.

**Figure 7:**
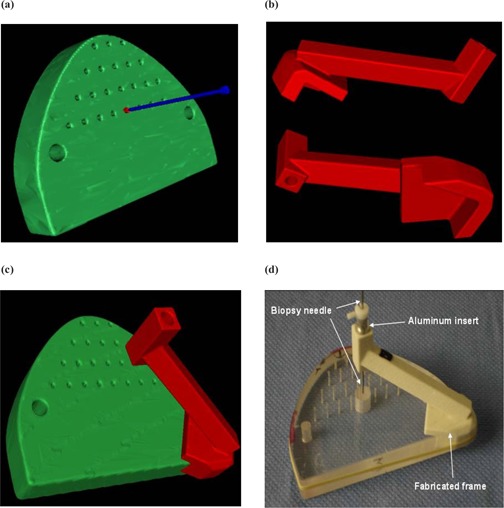
3D renderings that show the different steps of the fabrication of a positioning frame. (a) The surgical path is shown. The target point is the tip of the blue nail. The entry point is its head. (b) Two views of a virtual frame as designed by our program. (c) The virtual frame is shown positioned on the virtual model. (d) The fabricated frame is used to guide a biopsy probe to the target.

For all three selected surfaces, the same surgical path was defined by choosing two points: the entry point and the target point. To enable the assessment of alignment of defined surgical trajectory and attainable trajectory, the surgical path was taken as perpendicular to the phantom surface as possible. The target point is set at the center of the central hole at the surface of the phantom. The entry point is placed 60.0 mm from the target point along the axis of the small hole, perpendicular to the phantom surface, Fig. 7(a).

A biopsy needle was used as the surgical tool. We elected to incorporate a large diameter hole in the guide frame and to fabricate an aluminum insert to guide the needle (Fig. 7(d)). While the fabricated frame is compatible with the insertion of a biopsy needle, it is too soft to be used as a guide for a surgical drill bit. The aluminum insert provides not only a final guide hole that more precisely guides a surgical biopsy needle, but also one that is compatible with a surgical drill. The choice of aluminum over a harder material, such as stainless steel, was based on the ability to image with the guide in place, especially during initial clinical trials. The aluminum insert has its own length of 12.0 mm, which makes the total probe penetration into the guide equal to 72.0 mm.

### C. Fabrication of a custom reference frame

For each region of the phantom surface chosen for reference, we selected a subset of the initial image dataset that will be used by our program to build the frame. The reader can refer to the block “Frame builder” in the flowchart in Fig. 3. The building process is divided into two main steps. The first step creates (or designs) a binary image dataset that represents the shape of the final frame. Each voxel of this dataset is assigned a value 1 or 0, depending on whether it is inside or outside the frame limit. The second step uses the Marching Cube algorithm (threshold value of 0.5) to extract the contour of the frame from the binary image and decimates the resulting surface in a way similar to the extraction of the surface of the 3D model explained previously. The first step is the more sensitive part of the process because it determines the final shape of the frame. The entire step was performed at a voxel size of 0.3 mm. This value was chosen because (1) it was smaller than the resolution of the input image (0.35 mm in our case) and thus preserves the details contained in the CT scan, and (2) it was at least the accuracy of the 3DP (0.3 mm in our case); a better image resolution would have been useless because the 3DP would not reproduce such level of detail. The binary image (frame image) is built by first extracting the subset of the original image dataset that contains that portion of the phantom surface to be used for positioning the frame. Since the subset may not be aligned with the original image, the frame image dataset is obtained by linear interpolation between the voxels of the original image. The frame image is then smoothed using a Gaussian filter (same standard deviation as for the building of the model) and segmented by thresholding. At this point, the frame image is a binary image that represents two regions separated by the surface of the frame that will be in contact with the phantom. To provide a volume to the frame, the region that represents the inside of the phantom is dilated by 5 mm. The dilation is performed by convolution of a spherical kernel along the surface. All voxels reached by the sphere receive the value 1. This dilated image represents the outer surface of the frame 5 mm from the contact surface. The dilated image is then used as a mask applied to the segmented image in order to add the thickness of the frame. Following this, the guide is added to the image by changing all voxels of the image that belong to the volume of the guide to 1. The voxels are selected depending on the surgical path defined previously and based on the cylindrical shape of the aluminum insert. The frame image is now complete and can be used for extraction of the surface (step 2 described above). Examples of frames are shown in Figs. 7(b) and 7(c). Note that each step in the process will add a little uncertainty to the final result. While it is difficult to quantify the error for each step, we believe that the most important sources of error are related to the image resolution. The input image resolution will have an effect on the position of the contact surface, while the frame image (binary) resolution will affect the position of the guide. All other uncertainties (during image resampling, Gaussian filtration, and triangle decimation) should remain small compared to the image resolution.

**Figure 8:**
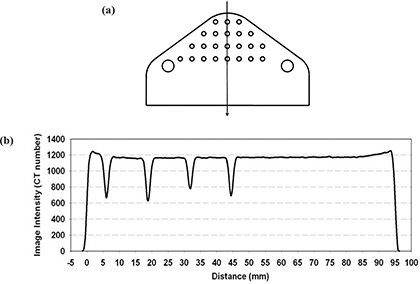
CT‐value profile along a straight line across the lexan phantom. (a) Scheme of the phantom showing the direction chosen to record the profile (the vertical arrow). (b) CT‐value profile along the straight line.

The exact surface of the object to be rendered is dependent upon the threshold used for segmentation. Figure 8 shows a diagram of the phantom with a graph of CT values along the indicated cross section. As can be seen, the CT numbers “ramp up” to the surface, a result of reconstruction artifact and partial volume effects. Each of the three frames was designed five times with a different threshold value: 400, 500, 600, 700, and 800. The 15 frames—three regions times five thresholds—were converted into an STL format and sent to the RP machine for fabrication.

**Figure 9:**
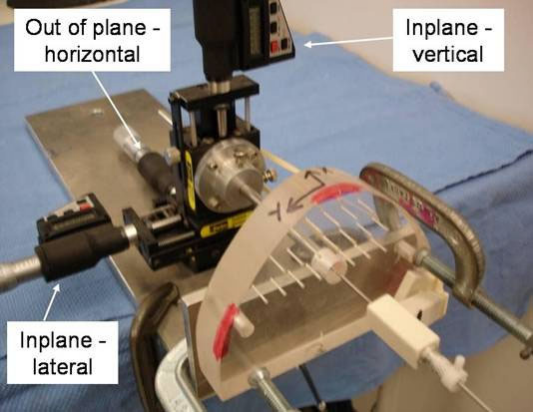
Device used to measure the position of the probe. The central rod is positioned by three orthogonal micrometers that are adjusted until the tip of the rod touches the tip of the probe. The micrometer positions are recorded and compared with the position of the planned target.

To test the accuracy of the targeting, the phantom was altered to allow the measurement of the tip of the biopsy needle's proximity to the center of the target hole. The hole was first drilled out to allow the introduction of a sharp tool. The tool was mounted onto a set of three orthogonal micrometers as shown in Fig. 9. Each micrometer is identified in the rest of this text by its position compared to the phantom: “in‐plane‐lateral,” “in‐plane‐vertical,” and “out of plane‐horizontal,” as shown in Fig. 9. The micrometers are on the positive side of the axis they represent. The tip reference was set to the defined target: the center of the hole and coincident to the surface of the phantom. The testing of a frame consisted of the user positioning the frame onto the phantom and attempting to find the best fit. The biopsy probe was introduced. The tip of the tool was then adjusted to touch the tip of the biopsy needle, and the resulting micrometer measurements were taken and compared with the reference position. The frame was removed and the process repeated. Four users each tested the 15 fabricated frames at their design positions. For each frame, the measurements made by the four users were averaged and a standard deviation was calculated.

### D. Test on a glass head phantom

**Figure 10:**
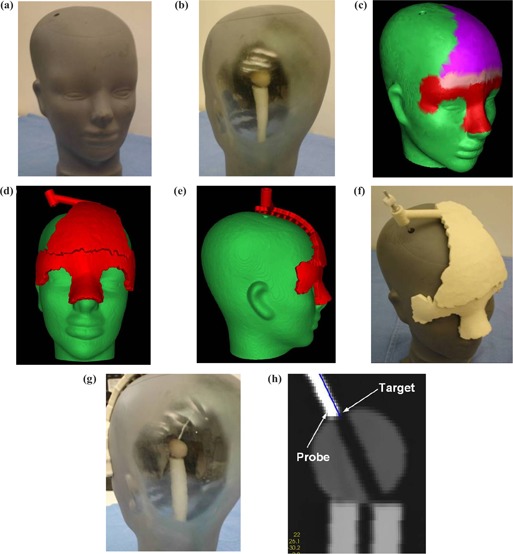
Glass‐head phantom used to test the design and fabrication of a reference frame. (a) The glass head is shown from the front. (b) A window allows one to view the target inside the head. (c) 3D model showing the reference surface selected by the user: in red where the frame is in contact with the skin and in purple where the frame incorporates spacers. (d) Frontal view of the phantom model with the virtual frame positioned. (e) Lateral view of the phantom model with the virtual frame, which shows the spacers that provide the gap for the patient's hair. (f) The reference frame is applied onto the phantom and the probe inserted into the guide. (g) The probe tip is shown inside the head next to the planned target. (h) 2D view showing the planned target (the tip of the blue line) and the tip of the probe (the white area on the image).

The design and fabrication process was repeated with a glass head phantom. The phantom was purchased from a local store and is made of standard recycled glass. It is shown in Fig. 10(a). A wooden sphere was incorporated inside the head, close to its center. The sphere was drilled with a hole along its diameter. The center of the most superior portion of the drill hole was defined as the target. A hole was also drilled through the glass head to allow a probe to reach the target from outside the head (Fig. 10(b)). The entry point was set along the axis of the hole, 120 mm from the target. The glass head was CT scanned using a Siemens Sensation 16 scanner. A head reconstruction protocol, an in‐plane resolution of 0.45 mm, and a slice thickness of 0.75 mm were used. The GUI was used to segment the head, identify the target and entry points, and fabricate the reference frame.

We developed a tool that allows the user to easily select the reference surface by “painting” the surface of the model. In the case of cranial surgery, it may not be desirable to shave the required reference surface. Designating such surfaces, the software introduces extended reference points, “teeth” (or spacers), to allow enough space for the patient's hair. Figure 10(c) shows the 3D model of the phantom after being painted by the user. The red paint shows where the frame is in contact with the skin. The purple paint shows where it is maintained away from the skin by spacers. The spacers are seen in Fig. 10(e). Using these extra features, the reference frame was designed, converted into STL format, and sent to the RP machine for fabrication.

The fabricated frame was positioned on the surface of the glass head, the aluminum insert was introduced into the frame, and a probe was set to a length of 132 mm (120 mm+12 mm for the aluminum insert) and introduced into the insert. The whole assembly (phantom, reference frame, insert, and probe) was CT scanned. This new scan was used to compare the position of the tip of the probe with the actual planned target. The planned target may not be exactly reidentified on the new scan at the same position as it was on the initial one (because this is done visually), so we decided to fuse (register) the new scan to the original dataset in order to transpose the position of the target into the new scan coordinate system. The fusion was performed using software that is routinely used in our department for clinical radiosurgery.^(14)^ The 3D reconstruction of the fused dataset was used to identify the position of the tip of the probe and to calculate its distance from the transposed target. The fusion may introduce an additional error to the final measurement, but we believe that this error would be smaller than the error produced by reidentifying the target position on a different scan.

## III. RESULTS AND DISCUSSION

### A. Phantom replica

**Figure 11:**
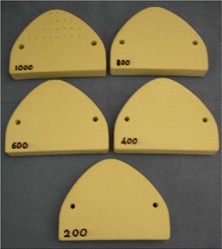
The five fabricated models. Each is built with a different threshold value indicated by the number printed on its surface.

The model of the lexan phantom was built five times with five different threshold values. The fabricated models are shown in Fig. 11. Only the replication with the threshold value of 1000 allows reproduction of the small holes. For lower thresholds, partial volume effects render the holes with insufficient contrast to be segmented, although the intersection of the hole and the phantom surface is still detectable at threshold values of 800 and 600. To estimate the accuracy of the replication of the phantom, we measured both the original phantom and the replicated models. The results are presented in Table 1. Note that a precision of 0.1 mm is provided. Due to the accuracy of the RP technology (0.3 mm at best), a more accurate measurement of the fabricated models would be meaningless. If we assume a smooth transition between air (CT number=0) and lexan (CT number=1200), the optimum threshold is expected to be 600. As can be seen in Table 1, the model fabricated with 600 does not always give the correct dimension. The errors shown in the table are the result of the measurement between two surfaces and suggest that the error for each surface is half the error mentioned above. This makes a maximum error of 0.25 mm (at threshold 600) for the position of a surface. This error is compatible with the accuracy of the RP technology.

**Table 1:** Measurements of the fabricated models compared to the phantom true measures. The numbers in parentheses represent the absolute errors from the phantom measurements.

	Threshold value					
Features measured	200	400	600	800	1000	Phantom
base (mm)	130.5 (+1.6)	130.0 (+1.1)	129.4 (+0.5)	128.8 (−0.1)	128.5 (−0.4)	128.9
height (mm)	96.6 (+0.9)	96.0 (+0.3)	95.6 (−0.1)	95.4 (−0.3)	95.3 (−0.4)	95.7
Thickness (mm)	24.5 (+0.1)	23.8 (+0.3)	23.3 (−0.2)	22.8 (−0.7)	22.3 (−1.2)	23.5
left hole (mm)	6.2 (−0.6)	6.4 (−0.4)	6.7 (−0.1)	7.0 (+0.2)	7.5 (+0.7)	6.8
right hole (mm)	5.8 (−1.0)	6.1 (−0.7)	6.7 (−0.1)	7.1 (+0.3)	7.6 (+0.8)	6.8

Both fabricated models at 400 and 800 also give reasonable results. A maximum error of 1.1 mm (0.55 mm for each surface) is seen for the 400 model and 0.7 mm (0.35 mm for each surface) for the 800 model. This suggests that the choice of the threshold value is not a critical parameter.

### B. Positioning frames

A total of 15 fixtures were fabricated, one for each of the five thresholds combined with one of the three positioning regions (“1,” “2,” and “3” in Fig. 6). An example of a virtual frame can be seen in Figs. 7(b) and 7(c). A fabricated frame is shown in Fig. 7(d) positioned on the surface of the phantom. Four persons were asked to assess the position of the probe using the micrometer apparatus described in section II.C. The averages and standard deviations over the four users are reported in Table 2. All numbers represent the distance between the tip of the biopsy needle and the planned target. A value of zero would indicate a tip right at the target. The first column in Table 2 identifies the fabricated frame. Columns 2, 3, and 4 are the averages (over the four measurers) and standard deviations obtained along the three micrometers. Column 5 shows the distance (average and standard deviation) between the needle tip and the target point. The needle position seems to go from the inside of the phantom surface to its outside as the threshold goes from 400 to 800, with the optimum threshold around 600.

**Table 2:** Differences between the position of the probe and the exact position of the target. The measurements were taken by four different persons, averaged, and the standard deviation calculated. Columns 2, 3, and 4 show the averages and standard deviations along each micrometer axis.

Frame‐threshold value	In‐plane: lateral (average±SD) (mm)	In‐plane: vertical (average±SD) (mm)	Out of plane: horizontal (average±SD) (mm)	Distance between probe and target (mm)
1 – 400	−0.88±0.89	−0.20±0.18	0.76±0.64	1.17±0.78
1 – 500	−0.94±1.11	0.35±0.18	0.01±0.43	1.00±1.04
1 – 600	−0.55±0.62	−0.26±0.31	−0.12±1.05	0.62±0.60
1 – 700	−0.34±0.78	0.23±0.69	−0.56±0.45	0.69±0.57
1 – 800	−1.27±1.22	−0.03±0.41	−1.08±0.36	1.67±0.96
2 – 400	0.37±0.65	0.16±0.24	0.95±0.38	1.03±0.42
2 – 500	−0.28±0.49	−0.11±0.17	0.93±0.56	0.98±0.56
2 – 600	−0.07±1.07	−0.51±0.62	0.07±1.09	0.52±0.65
2 – 700	0.01±0.65	0.25±0.33	0.21±0.34	0.32±0.33
2 – 800	0.13±0.99	−0.21±0.48	−0.45±0.32	0.51±0.43
3 – 400	0.20±0.19	0.77±0.28	0.48±0.20	0.93±0.26
3 – 500	0.11±0.28	0.66±0.45	0.32±0.36	0.74±0.43
3 – 600	−0.17±0.31	0.80±0.26	−0.08±0.24	0.82±0.26
3 – 700	−0.20±0.32	0.32±0.43	−0.11±0.08	0.39±0.38
3 – 800	−0.06±0.32	0.84±0.23	−1.25±0.03	1.51±0.13

Table 2 shows a second important consequence of the measurement results. The standard deviations remain small for frame 3, whereas they can be very large for frames 1 and 2. This is a consequence of the geometry of the positioning surface. Frame 3 (located at the corner of the phantom), when positioned on the phantom surface, seems very stable and allows little room for error. On the other hand, frames 1 and 2 have a contact surface with a gentle slope that does not provide a unique fit on the phantom and explains the user's difficulty in finding the correct position. As a consequence, we infer that the selection of the contact surface is the most critical part of the overall process. Furthermore, if we choose 600 for the threshold, Table 2 shows that the maximum average distance in column 5 is 0.82 mm from the target.

Table 3 represents the average and standard deviation of the distance to the target (column 5 of Table 2) when the distances are grouped by threshold value. It can be seen that better results are obtained with the 600 and 700 models, which is compatible with the study on phantom replicas (results in Table 1).

**Table 3:** Distance between the probe and the exact position of the target per threshold value. Each value represents the mean and standard deviation for the distance to target (Table 2, column 5) from the three frames built with the same threshold value.

Threshold	Distance between probe and target(mm)
400	1.04±0.12
500	0.91±0.14
600	0.65±0.15
700	0.47±0.20
800	1.23±0.63

### C. Glass head phantom

A reference frame was fabricated to access a target inside a glass head. Figures 10(d) and 10(e) show the virtual frame positioned on the surface of the 3D model. The contact surface covers a large area and uses the complex region of the nose to ensure a unique fit of the frame. In Fig. 10(f), the fabricated frame is placed on the glass head, and the probe is inserted into the guide. Figure 10(g) shows the tip of the probe next to the sphere.

Figure 10(h) shows a 3D reconstruction of the CT scan of the assembly head, frame, insert, and probe after it was registered to the initial image. The blue line represents the tip of the planned trajectory. This picture allows one to compare the position of the probe with the planned target. The tip of the probe was visually localized within the image dataset using the orthogonal cross‐sectional images and the 3D rendering of the probe tip. The probe position was recorded and the distance to the transposed target calculated to 1.7 mm. The error is larger than the errors measured in the previous experiment. There are two reasons for this difference:

- The probe was longer in this experiment (132 mm compared to 72 mm used for the lexan phantom test), and for an identical angular error, the deviation at the tip of the probe is expected to be larger.
- The measurement technique used in this experiment is based on a CT scan that has a resolution of 0.45 mm. In the previous experiment, the measurement was performed with micrometers that are probably one order of magnitude more accurate. The two images were also fused, and this process introduces its own additional uncertainty.

Note that these errors are measurement errors and cannot be considered part of the overall technique accuracy. They are included in the final error estimation, so the actual accuracy of the technique may well be better than the 1.7 mm found.

### D. Clinical application

Application of this technology is currently being used in an IRB‐approved trial for the placement of pediatric ventriculo‐peritoneal shunts. One day prior to surgery, a customized RP guide is built after the surgeon chooses a target and entry point on the computer model. In the operating room, the guide is placed on the patient. While a second person holds the frame firmly in place, the surgeon introduces the probe into the guide. The target and trajectory provided by the guide are verified against a framed or frameless navigation system. The coordinates of the target provided by the customized guide are compared to the coordinates provided by the navigation system. The accuracy of the prototype guide is judged by the degree of congruence of the coordinates. Note that in clinical cases, the head of the patient is often supported by the couch of the scanning device during the imaging session and looks attached to the supporting device in the scan. This would be a problem when trying to segment the skin contour. Although the back of the head is not often used for designing the positioning frame, some very low‐density materials (such as towels or foam blocks) should be introduced between the couch and the patient head to avoid the problem.

## IV. CONCLUSIONS

In this study, we investigated the use of RP technology to replicate a tissue‐like phantom from a CT scan and to use the image dataset information to fabricate a customized positioning fixture for guiding a biopsy needle to a surgical target. First, the choice of a threshold value for segmenting the image dataset is not critical. A value midway between the CT numbers of the two segmented materials provides acceptable results, and any attempt to find a better segmentation technique would require an improved build resolution of the currently used RP technology. For clinical cases, the value routinely used for skin segmentation is 500, the value chosen for our IRB trails.

Concerning the fabrication of positioning surgical reference frames, a similar conclusion can apply. Because of the sharp transitions between air and the patient's surface, the effect of the threshold choice on the system accuracy is not of critical importance. The major issue in designing the reference frame is to select the surface that will provide a unique fit during surgery. The test with the phantom showed that it can be difficult for the surgeon to position the frame correctly if the surface does not provide a unique fit. If an unambiguous geometric surface can be identified by the surgeon, the accuracy and precision superior to previously documented frame based procedures are obtainable. In our current IRB trial we found that facial features, similar to those used with the glass head phantom, provide the required degree of fit to support acceptable accuracy. One of our future objectives is to provide the surgeon with an automatic surface selection to reduce the planning time and to eliminate human errors. Several solutions are foreseen. One can involve growing a surface around a user‐defined point until the whole surface provides a unique fit and ensures stability when applied to the patient's skin. A second solution is to determine a predefined surface (stable and providing a unique fit) from a reference case (model) and to transpose the reference surface onto the clinical case. By morphing the model frame onto the patient anatomy, it should be possible to provide a customized frame that keeps stability and uniqueness of fit. Future investigations are needed.

Most targets in the brain are accessible within less than 175 mm (including the length of the guide and a gap between the skin and the guide), so we expect a maximum error of less than 2 mm for any point within the head. The level of accuracy demonstrated is sufficient to satisfy the criteria of equivalence to commercially available frameless optically guided procedures. As mentioned in section II, the major sources of error for the process are found in the original image resolutions, the image‐processing steps (resampling, Gaussian filtering, and triangle decimation), the frame image resolution (used to design the guide), and the frame fabrication. All these steps are subject for improvement in the future.

The success in phantom and in initial IRB trials supports a more extensive evaluation of the use of rapid prototyping models for guidance of shunts, biopsies, and localization of craniotomies and identifying the trajectories for all currently available image‐guided procedures. Such investigations under IRB protocols are currently underway.

## ACKNOWLEDGMENT

This work was supported by grant #5R01EB2573‐3 from the National Institutes of Health NIH‐NIBIB.

## References

[1] Maciunas RJ , Galloway RL , Latimer JW . The application accuracy of stereotactic frames. Neurosurgery 1994; 35 (4): 682–694. 7808612 10.1227/00006123-199410000-00015

[2] Galloway RL , Maciunas RJ , Latimer JW . The accuracies of four stereotactic frame systems: An independent assessment. Biomed Instrum Technol. 1991; 25 (6): 457–460. 1777768

[3] Yan X , Gu P . A review of rapid prototyping technologies and systems. Comp Aided Design. 1996; 28 (4): 307–318.

[4] Hull C , Feygin M , Baron Y , et al. Rapid prototyping: Current technology and future potential. Rapid Prototyping J. 1995; 1 (1): 11.

[5] Wohlers T . Future potential of rapid prototyping and manufacturing around the world. Rapid Prototyping J. 1995; 1 (1): 4.

[6] Pham DT , Gault RS . A comparison of rapid prototyping technologies. Int J Machine Tools Manufacture. 1998; 38 (10–11): 1257–1287.

[7] Sachs E , Cima M , Cornie J . Three dimensional printing: Rapid tooling and prototypes directly from a CAD model. Ann CIRP. 1990; 39 (11): 210–214.

[8] Crump SS . The extrusion process of fused deposition modeling. Dayton (OH): Proceedings of the third international conference on rapid prototyping 1992; 91–102.

[9] Comb JW , Priedeman WR , Turley PW . FDM technology process improvements. Proceedings of the fifth solid freeform fabrication symposium. Austin (TX): University of Texas Press 1994; 42–91.

[10] Konig W , Celi I , Noken S . Stereolithography process technology. Proceedings of the 3rd European conference on rapid prototyping. Nottingham (UK): University of Nottingham Press 1994; 191–208.

[11] Chalasani K , Jones L , Roscoe L . Support generation for fused deposition modeling. Proceedings of the sixth annual solid freeform fabrication symposium. Austin (TX): University of Texas Press; 1995; 229–241.

[12] Lee SJJ , Sachs E , Cima M . Layer positioning accuracy in powder‐based rapid prototyping. Rapid Prototyping J. 1995; 1 (1): 24.

[13] Lorensen WE , Cline HE . Marching cubes: A high resolution 3D surface construction algorithm. Comp Graph. 1987; 21 (4): 163–169.

[14] Friedman WA , Bova FJ . The University of Florida radiosurgery system. Surg Neurol. 1989; 32 (50): 334–342. 2683164 10.1016/0090-3019(89)90135-3

